# EGCG Inhibits Proliferation and Induces Apoptosis Through Downregulation of SIRT1 in Nasopharyngeal Carcinoma Cells

**DOI:** 10.3389/fnut.2022.851972

**Published:** 2022-04-25

**Authors:** Shisheng Jiang, Chaoming Huang, Guodong Zheng, Wei Yi, Bo Wu, Junyuan Tang, Xiawen Liu, Biyun Huang, Dan Wu, Tingdong Yan, Mingxi Li, Chunpeng Wan, Yi Cai

**Affiliations:** ^1^Key Laboratory of Molecular Target & Clinical Pharmacology, The State & NMPA Key Laboratory of Respiratory Disease, School of Pharmaceutical Sciences, Fifth Affiliated Hospital, Guangzhou Medical University, Guangzhou, China; ^2^School of Pharmacy, Nantong University, Nantong, China; ^3^Research Center of Tea and Tea Culture, College of Agronomy, Jiangxi Agricultural University, Nanchang, China

**Keywords:** EGCG, nasopharyngeal carcinoma, SIRT1, p53, apoptosis

## Abstract

Epigallocatechin-3-gallate (EGCG), a frequently studied catechin in green tea, has been shown involved in the anti-proliferation and apoptosis of human nasopharyngeal carcinoma (NPC) cells. However, the underlying molecular mechanism of the apoptotic effects of EGCG has not been fully investigated. Recent literature emphasized the importance of Sirtuin 1 (SIRT1), an NAD^+^-dependent protein deacetylase, in regulating cellular stress responses, survival, and organismal lifespan. Herein, the study showed that EGCG could significantly inhibit cell proliferation and promote apoptosis of 2 NPC (CNE-2 and 5-8F) cell lines. Moreover, it was also found that SIRT1 is down-regulated by EGCG, and the SIRT1-p53 signaling pathway participates in the effects of EGCG on CNE-2 and 5-8 F cells. Taken together, the findings of this study provided evidence that EGCG could inhibit the growth of NPC cell lines and is linked with the inhibition of the SIRT1-p53 signaling pathway, suggesting the therapeutic potential of EGCG in human NPC.

## Introduction

Nasopharyngeal carcinoma (NPC) is a malignant tumor with a high incidence rate in the Southeast Asia and Southern China (1, 2). Currently, radiotherapy combined with chemotherapy is routinely used to control early disease progression. Due to the insidious location of NPC and lack of obvious early symptoms, over 70% of NPC patients present with locally advanced or metastatic lesions at the time of diagnosis (3). The effect of radiotherapy alone is unsatisfactory, so adjuvant chemotherapy is essential. As first-line chemotherapeutic drugs for NPC, cisplatin, and paclitaxel can significantly improve NPC's therapeutic efficacy. But the larger doses of cytotoxic drugs often lead to severe toxic side effects by inducing cancer multi-drug resistance and, hence, are declared the main treatment failures (4). Therefore, it is necessary to find high-efficacy with lower toxicity chemotherapeutic agents to improve the patients' clinical efficacy and high survivorship.

Natural products have historically contributed to pharmacotherapy, especially for cancer and infectious diseases (5, 6). (-)-Epigallocatechin-3-gallate (EGCG), is a polyphenolic component extracted from green tea, has been demonstrated to exhibit a variety of health benefits such as anti-tumor, anti-oxidant, anti-inflammatory, cardiovascular protection, and neuroprotection (7, 8). The recent studies revealed that EGCG inhibited proliferation and improved the sensitization of NPC cells to radiotherapy, which suggested the therapeutic potential of EGCG on NPCs (9, 10). However, little is known about potential targets for EGCG-induced inhibition in NPC cells.

SIRT1 is a prominent and extensively studied member of the sirtuins family of the mammalian class III histone deacetylases heavily implicated in healthspan and aging (11). The vast literature demonstrated that SIRT1 expression increased in most human cancers, especially, prostate cancer, primary colon cancer, acute myeloid leukemia, and squamous epithelial cell carcinoma (12). Studies have also reported that the expression levels of SIRT1 are linked with cancer invasion/metastasis and drug resistance, which in turn affect the prognosis of cancers (13). Recently, literature has focused that SIRT1 can promote the proliferation, migration, and lipid metabolism in NPC cells, suggesting that SIRT1 is an attractive target to treat NPCs (14). However, it is not known whether the SIRT1-dependent signaling pathway participates in the effect of EGCG on NPC cells. Therefore, this study aimed to investigate the effect of EGCG on the growth of NPC (CNE-2 and 5-8F) cell lines and determine whether the SIRT1 signaling is involved in this phenomenon or not.

## Regents and Methods

### Reagents

Human NPC cell lines (CNE-2 and 5-8F) were purchased from cell bank of the Chinese Academy of Sciences (Shanghai, China). RPMI 1,640 medium and fetal bovine serum (FBS) was purchased from Gibco (Logan, UT, USA). Rabbit anti-caspase 3 (#9,662), Rabbit anti-α-tubulin (#21,44S), Rabbit anti-Apaf-1 (#89,69S), Rabbit anti-caspase 9 (#95,08S), Rabbit anti-Acetyl-p53 (Lys382) (#25,25S), and Rabbit anti-SIRT1 (#2,493) were purchased from Cell Signaling Technology (Boston, MA, USA). EGCG (purity > 95%) was purchased from Sigma Aldrich and dissolved in distilled water at 100 μM, and stored at −20°C until dilution before use. The cell counting kit 8 (CCK8) was purchased from DOJINDO (Japan). The terminal deoxynucleotidyl transferase (TDT)-mediated dUTP nick end labeling (TUNEL) assay kit was purchased from the Nanjing KeyGen Biotech Co., Ltd. (Nanjing, China). The BCA protein assay kit was purchased from Thermo Fisher Scientific (Chicago, IL). Super signal chemiluminescent substrate (ECL) was obtained from Thermo Scientific (Waltham, MA, USA).

### Cell Culture

Cells (CNE-2 and 5-8F) were grown in RPMI-1640 culture medium supplemented with 10% FBS, 100 u/ml penicillin, and 100 mg/ml streptomycin at 37°C in humid incubator contains 95% air and 5% CO_2_. The medium was changed every other day. When the culture reaches 70~90% confluence, cells are subcultured at a split ratio of 1:3. Before treating with EGCG, cells were cultured in FBS-free for 12 h.

### CCK-8 Analyses

Cells (CNE-2 and 5-8F) were seeded at 1 × 10^4^/well in 96-well plates. After being cultured in a complete medium for 24 h, the cells changed to an FBS-free medium for another 12 h at 37°C. Then, the cells were treated with EGCG at the indicated concentration (0, 10, 20, and 40 μm) or with 40 μm after overexpression of SIRT1 for 24 h. A total of 10 μl of CCK-8 solution was added to each well and incubated for another 1 h, followed by reading the absorbance at 450 nm using a Multi-Volume Spectrophotometer system (BioTek Instruments, Inc., USA).

### Real-Time PCR

Total RNA from CNE-2 cells was extracted with TRIzol. cDNA was generated using the PrimeScript™ RT reagent kit (Takara Bio Inc., Japan). The mRNA expression levels were measured using a quantitative PCR Kit (Takara Biotechnology) by the iCycler iQ system (Bio-Rad). Amplification conditions were 15 min at 95°C, followed by 40 cycles of 30 s at 95°C, 1 min at 55°C, and 30 s at 72°C. Human-specific primers (Supplementary Table S1) for Bax, Bcl-2, and GAPDH were synthesized by Invitrogen. The GAPDH was used as endogenous control. Each PCR amplification was performed in triplicate.

### TUNEL Assay

TUNEL analyses were performed as described previously (15). Cells were fixed with 4% formaldehyde in PBS at room temperature for 30 min. After two washes in PBS, the cells were permeabilized using 0.2% TritonX-100 in PBS for 5 min. Then, the cells were labeled with Biotinylated dUTP and TDT enzymes in a humidified box at 37°C for 1 h, followed by incubation with streptavidin-fluorescein for 30 min. Finally, cells were counterstained with 4',6-diamidino-2-phenylindole (DAPI) at room temperature for 10 min before observation under a microscope. Cells were only labeled as being TUNEL positive and expressed as a percentage of the total nuclei.

### Western Blot Analysis

Western blot assays were performed as described previously (16). Total protein was exacted from CNE-2 or 5-8F cells using RIPA lysis buffer, and the protein concentration was evaluated using a BCA protein assay kit (Thermo Fisher Scientific). The same amount of protein (20 μg per lane) was separated by SDS-PAGE gel electrophoresis and then transferred to the PVDF membrane (Millipore). After blocking 5% nonfat milk, the membranes were incubated with primary antibodies overnight at 4°C. Next, membranes were incubated with appropriate horseradish peroxidase (HRP)-labeled second antibodies for 1 h at room temperature. At last, the blotted membranes were visualized by the enhanced chemiluminescent (ECL) method, and molecular band intensity was determined by densitometry. α-tubulin was used as endogenous control. The intensities of the blots were quantified by densitometry using the image lab analysis system and NIH image J software.

### SIRT1 Histone Deacetylase Activity Assay

The histone deacetylase activity assay was performed as described previously (16). Total proteins, extracted from CNE-2 or 5-8F cells, were immunoprecipitated with anti-SIRT1 antibody and protein G agarose beads (Pierce). After incubation overnight at 4°C, the precipitates were transferred into a new tube. The reaction was carried out by mixing 15 μl of precipitates and 35 μl reaction mixture (20 μM fluorosubstrate peptide, 800 μM NAD, 0.25 mAU/ml lysylendopeptidase, 1 μm trichostatin A and 50 mM Tris-HCl) according to the manufacturer's instructions (CycLex, Ina). Then, the intensity of fluorescence was detected by microtiter plate fluorometer (excitation filter: 490 ± 10 nm, emission filter: 530 ± 15 nm) and normalized by the protein concentration.

### Plasmid Transfection and RNA Interference

For SIRT1 overexpression and RNA interference, CNE-2 and 5-8F cell lines were transfected with flag-empty, flag-SIRT1, siSIRT1, and negative control siRNA, respectively, using Lipofectamine 2000 (Invitrogen) according to the procedure described in our previous study (17). At 48 h after transfection, real-time PCR, western blotting, and fluorometric assay kit were used to evaluate the transfection efficiency. A total of three different duplex siRNAs (Supplementary Table S2) for SIRT1 (siSIRT1-1, siSIRT1-2, and siSIRT1-3) and negative control siRNA were purchased from Genepharma (Shanghai, PR China).

### Molecular Docking

According to a previous study, the molecular docking was carried out using Discovery Studio 3.1. The crystal structure of SIRT1 was obtained from the Protein Data Bank (PDB ID: 4KXQ). We first used the protein preparation wizard to deal with the protein structure and reassigned the bone orders. Then, we minimized the sampled hydrogens to fine tune the hydrogen-bonding network. Finally, a restrained minimization was conducted by applying the CHARMm force field. In addition, EGCG was processed through increasing explicit hydrogen atoms and using the CHARMm force field and geometry optimization with the prepared ligands module of Discovery Studio 3.1. The docking grid was generated with its center set to the native ligand, and the grid size was set similarly (the search grid of binding site was identified as center x: 32.225016, center y: −16.502403, and center z: 9.395139 with the radius of 10.297739). After completing the aforementioned preparatory work, EGCG was performed to docking at the extra precision (XP) level.

### Statistical Analysis

Data are presented as mean ± standard error (SE). Statistical analyses between two groups were performed by unpaired Student's *t*-test. Differences among groups were tested by one-way ANOVA. In all the cases, differences were considered statistically significant with *P* < 0.05.

## Results

### Effect of EGCG on Proliferation and Apoptosis of NPC Cell Lines

**Figure 1:**
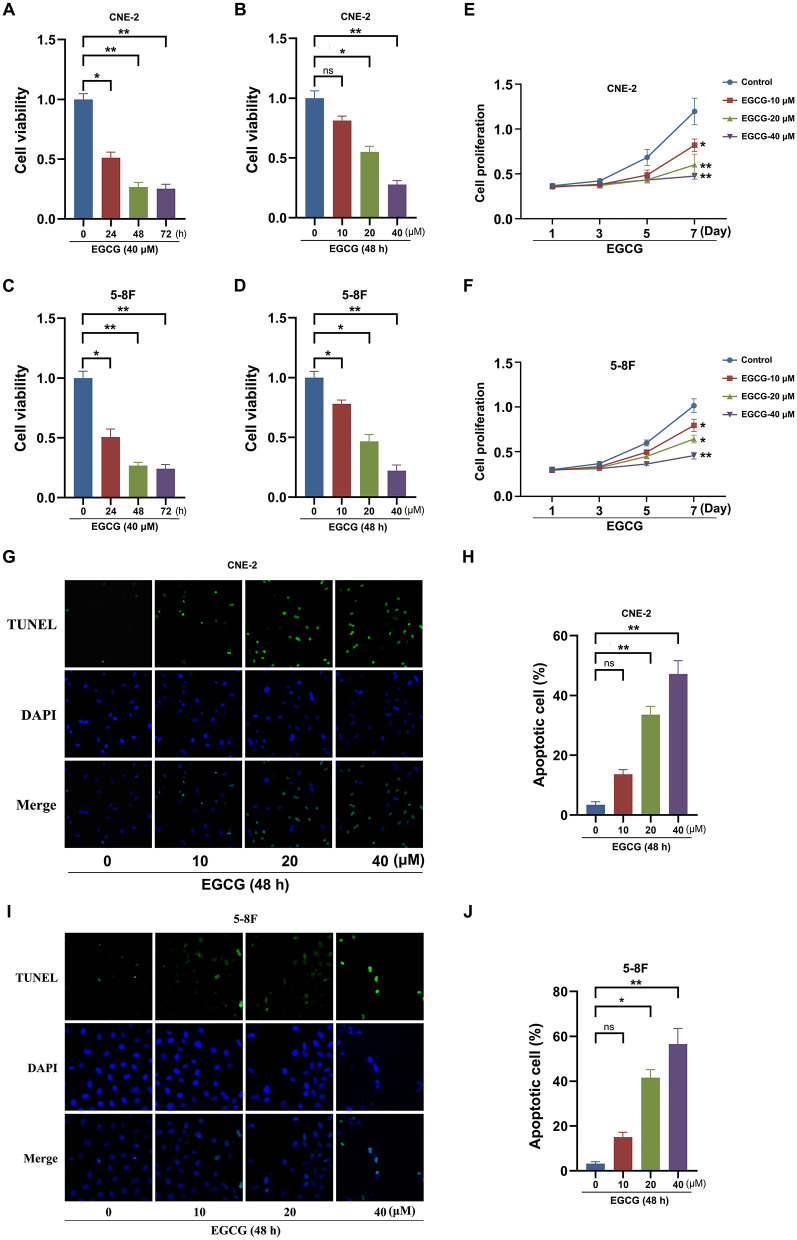
Effect of EGCG on proliferation and apoptosis of NPC cell lines. CNE-2 and 5-8F cells were treated with various concentrations of EGCG for 48 h or with 40 μM EGCG for indicated time points. The cell viability **(A–D)** and proliferation kinetics **(E,F)** were detected by CCK8 assay. Cells were treated with various concentrations of EGCG for 48 h. **(G–J)** The cell apoptosis was detected by tunnel assay. *P < 0.05 and **P < 0.01 vs. the group without treatment, *n* = 5. ns = no significance which means no statistical difference.

**Figure 2:**
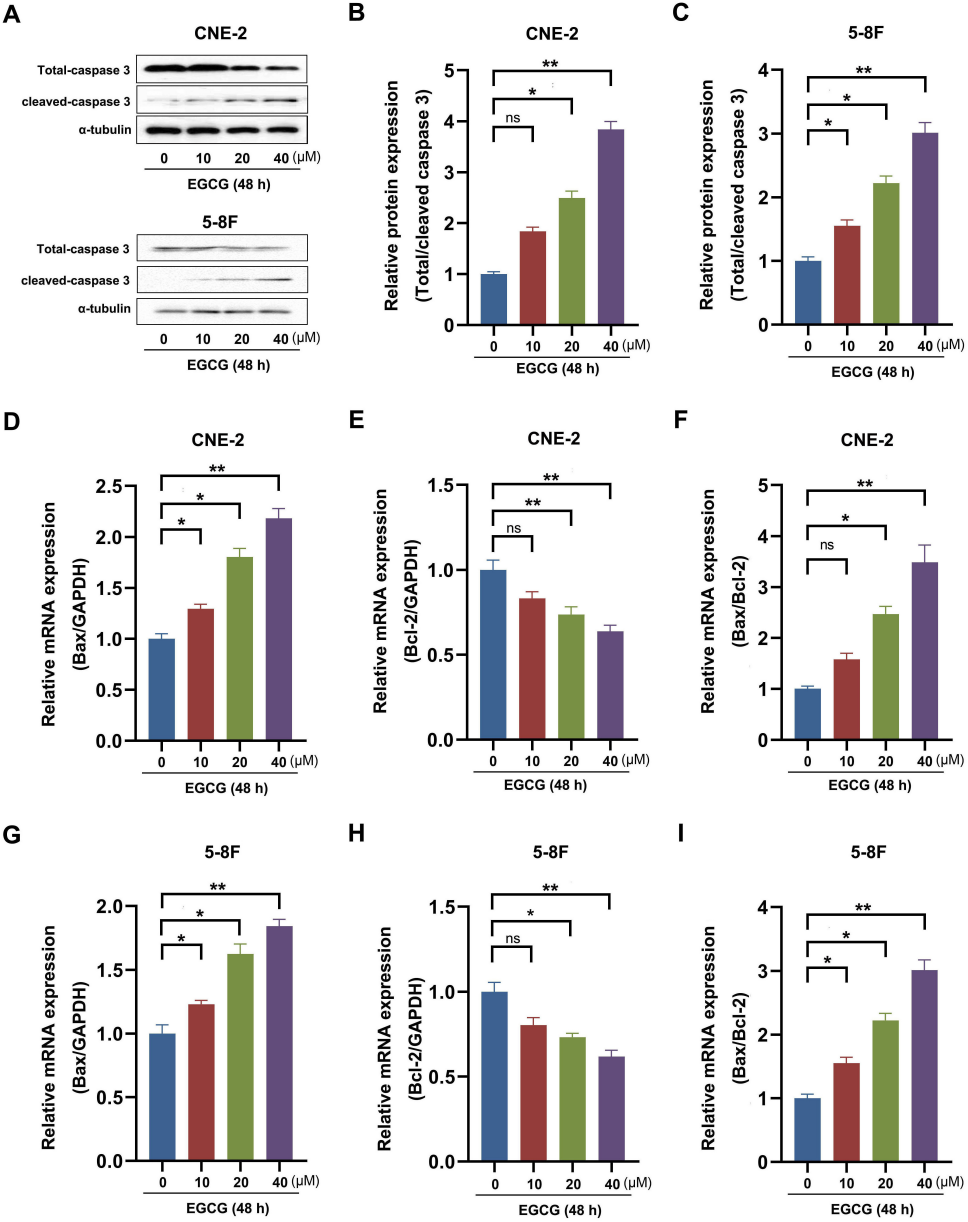
Effect of EGCG on apoptosis-related genes expression in NPC cell lines. CNE-2 and 5-8F cells were treated with various concentrations of EGCG for 48 h. **(A–C)** The protein expression of caspase 3 was analyzed by Western Blotting. **(D–I)** The mRNA expression of Bax and Bcl-2 was determined by real-time PCR. *P < 0.05 and **P < 0.01 vs. the group without treatment, *n* = 5. ns = no significance which means no statistical difference.

To investigate the effect of EGCG on the proliferation of NPC cell lines, CNE-2 and 5-8F cells were incubated with various concentrations of EGCG at different times. And the cell viability was assessed by CCK8 assay. As shown in Figures 1A–D, EGCG inhibited NPC cell lines (CNE-2 and 5-8F) viability in a dose- and time-dependent manner. We further tested the proliferation kinetics of NPC cells and showed that EGCG treatment could significantly inhibit cell proliferation in CNE-2 and 5-8F cell lines (Figures 1E,F). Then, we detected the effect of EGCG on the apoptosis of NPC cells. As shown in Figures 1G–J, EGCG-induced apoptosis in NPC cell lines. Meanwhile, EGCG caused caspase-3 cleavage (Figures 2A–C). Moreover, EGCG also resulted in a dose-dependent increase in the Bax/Bcl-2 ratio that favors apoptosis in NPC cell lines (CNE-2 and 5-8F) (Figures 2D–I).

### Effect of EGCG on Expression and Activity of SIRT1 in NPC Cell Lines

**Figure 3:**
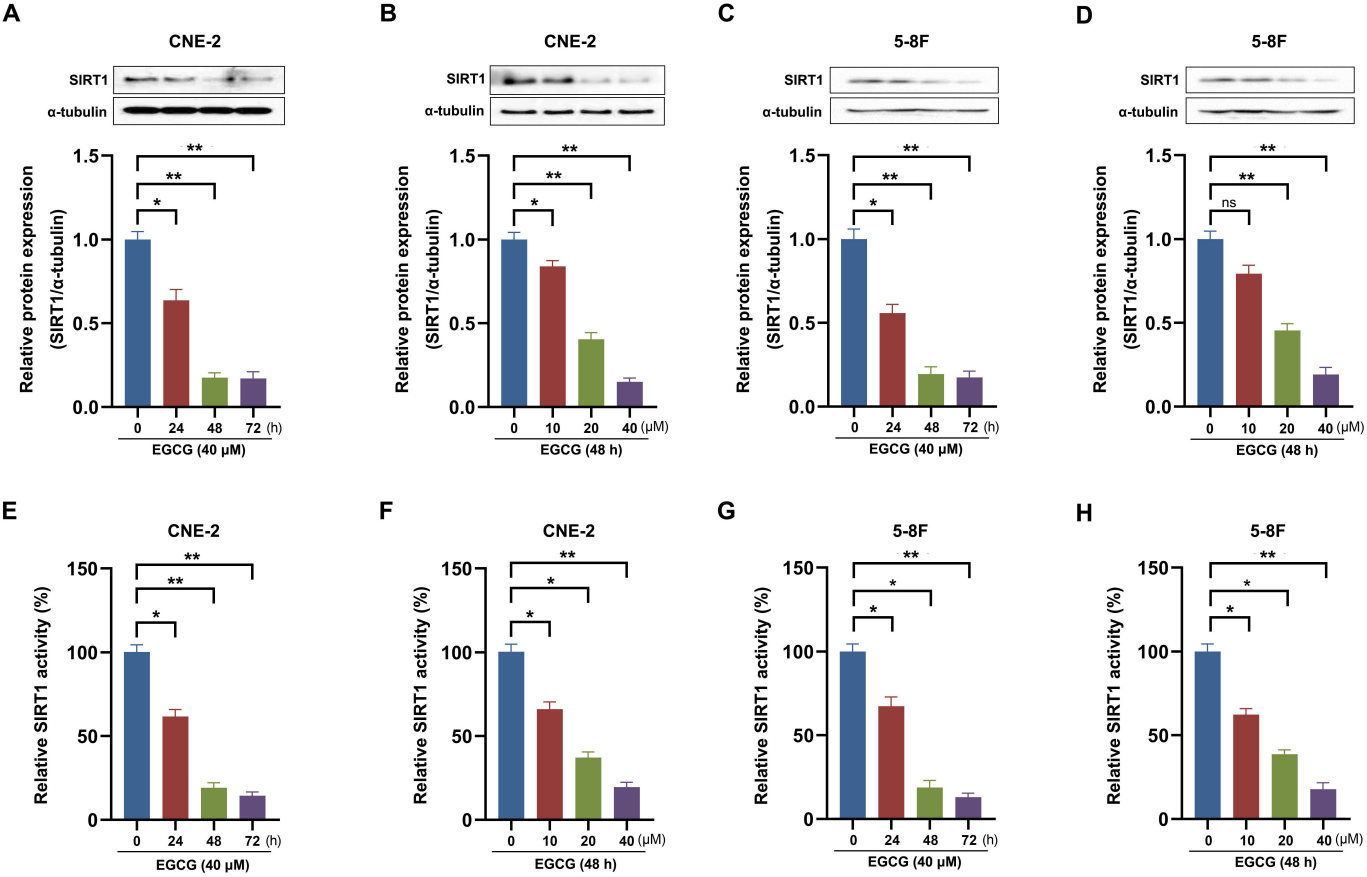
Effect of EGCG on expression and activity of SIRT1 in NPC cell lines. CNE-2 and 5-8F cells were treated with various concentrations of EGCG for 48 h or with 40 μM EGCG for indicated time points. The protein expression **(A–D)** and deacetylase activity **(E–H)** of SIRT1 were detected by Western Blotting and deacetylase fluorometric assay kit, respectively. *P < 0.05 and **P < 0.01 vs. the group without treatment, *n* = 5. ns = no significance which means no statistical difference.

Previous studies have demonstrated that SIRT1 activation promoted NPC cells' proliferation, migration, and lipid metabolism. It indicated that SIRT1 might participate in the effect of EGCG on NPC cell lines. To elucidate the potential molecular mechanisms of EGCG on NPC cells *in vitro*, we checked the protein level of SIRT1. As shown in Figures 3A–D, EGCG could inhibit the protein expression of SIRT1 in a time- and dose-dependent manner. Then, we further assessed the histone deacetylase activity of SIRT1 and found that EGCG inhibited the enzyme activity of SIRT1 in NPC cell lines (Figures 3E–H).

### SIRT1 Participate in the Regulation of EGCG on Proliferation and Apoptosis in NPC Cell Lines

**Figure 4:**
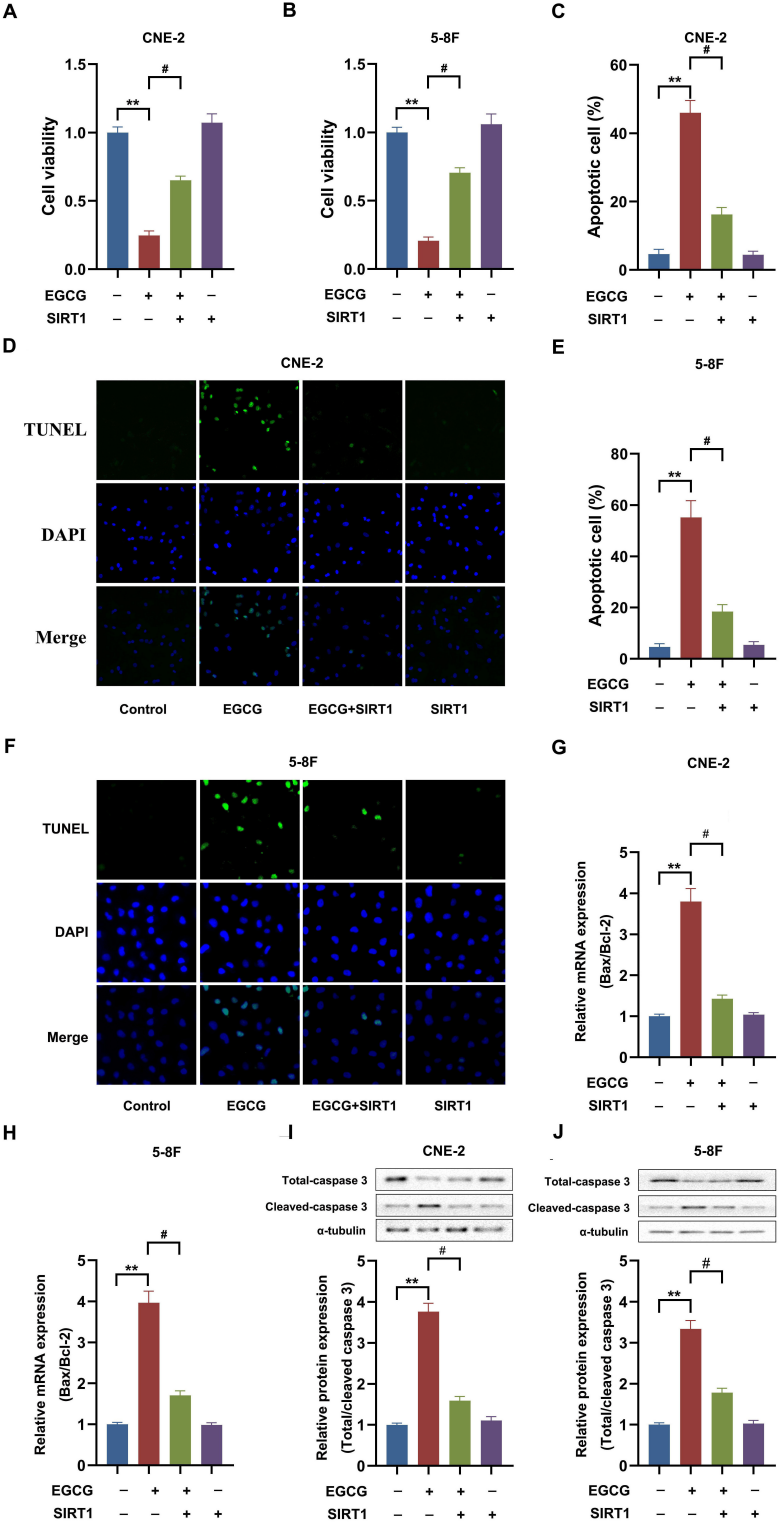
SIRT1 overexpression ameliorated the effect of EGCG on proliferation and apoptosis in NPC cell lines. Cells transfected with or without SIRT1 were treated with 40 μM EGCG for 48 h. **(A,B)** The cell viability was detected by CCK8 assay. **(C–F)** The cell apoptosis was detected by tunnel assay. **(G,H)** The mRNA expression of Bax and Bcl-2 were determined by real-time PCR. **(I,J)** The protein expression of caspase 3 was analyzed by Western Blotting. **P < 0.01 *vs*. the group without treatment, ^#^*P* < 0.05 vs. the group without treatment with EGCG alone, *n* = 5.

**Figure 5:**
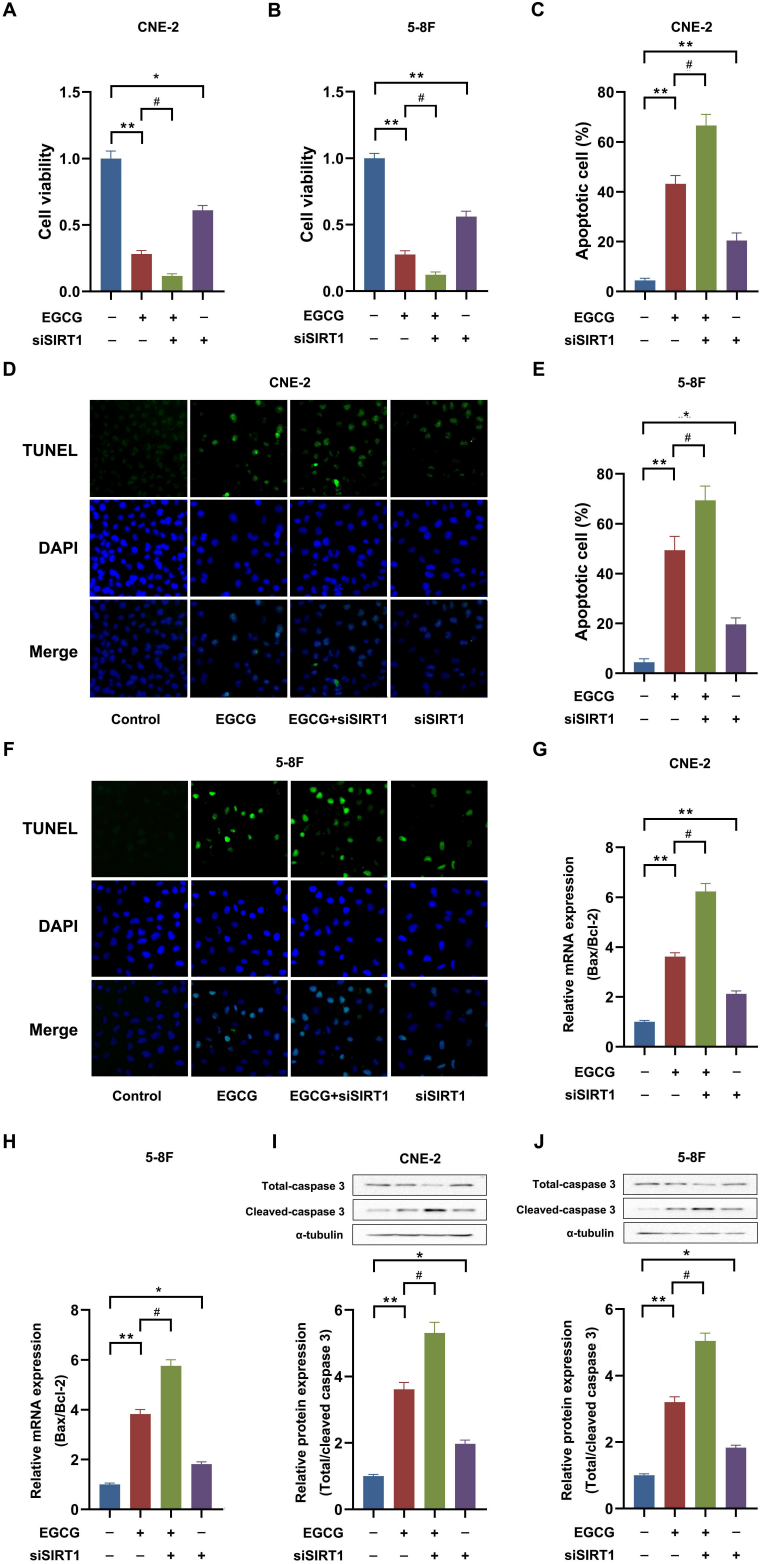
SIRT1 knockdown aggregated the effect of EGCG on proliferation and apoptosis in NPC cell lines. Cells transfected with or without siSIRT1 were treated with 40 μM EGCG for 48 h. **(A,B)** The cell viability was detected by CCK8 assay. **(C–F)** The cell apoptosis was detected by tunnel assay. **(G,H)** The mRNA expression of Bax and Bcl-2 were determined by real-time PCR. **(I,J)** The protein expression of caspase 3 was analyzed by Western Blotting. *P < 0.05 and **P < 0.01 *vs*. the group without treatment, ^#^*P* < 0.05 vs. the group without treatment with EGCG alone, *n* = 5.

To examine the role of SIRT1 in EGCG-induced apoptosis, cells were transfected with SIRT1. Overexpression of SIRT1 significantly increased the expression and activity of SIRT1 in CNE-2 and 5-8F cells (Supplementary Figure S1). Then, we checked the cell viability and apoptosis in NPC cell lines. As shown in Figures 4A–F, EGCG treatment inhibited the proliferation and induced apoptosis in CNE-2 and 5-8F cells, which could be attenuated by preincubation with SIRT1 overexpression. Moreover, when SIRT1 was overexpressed, EGCG failed to increase the Bax/Bcl-2 ratio (Figures 4G,H) and caspase-3 cleavage (Figures 4I,J). Then, we tested the effect of SIRT1 siRNA interference on proliferation and apoptosis of NPC cells induced by EGCG. When the cells were transfected with siSIRT1, the expression and activity of SIRT1 were decreased significantly (Supplementary Figure S2). We further measured the cell viability and apoptosis in NPC cell lines. As shown in Figure 5, knockdown of SIRT1 aggravated the cell proliferation inhibition and apoptosis in NPC cell lines (Figures 5A–F), as well as the Bax/Bcl-2 ratio (Figures 5G,H) and caspase 3 cleavage (Figures 5I,J).

### Effect of EGCG on p53-Dependent Apoptotic Pathways in NPC Cell Lines

**Figure 6:**
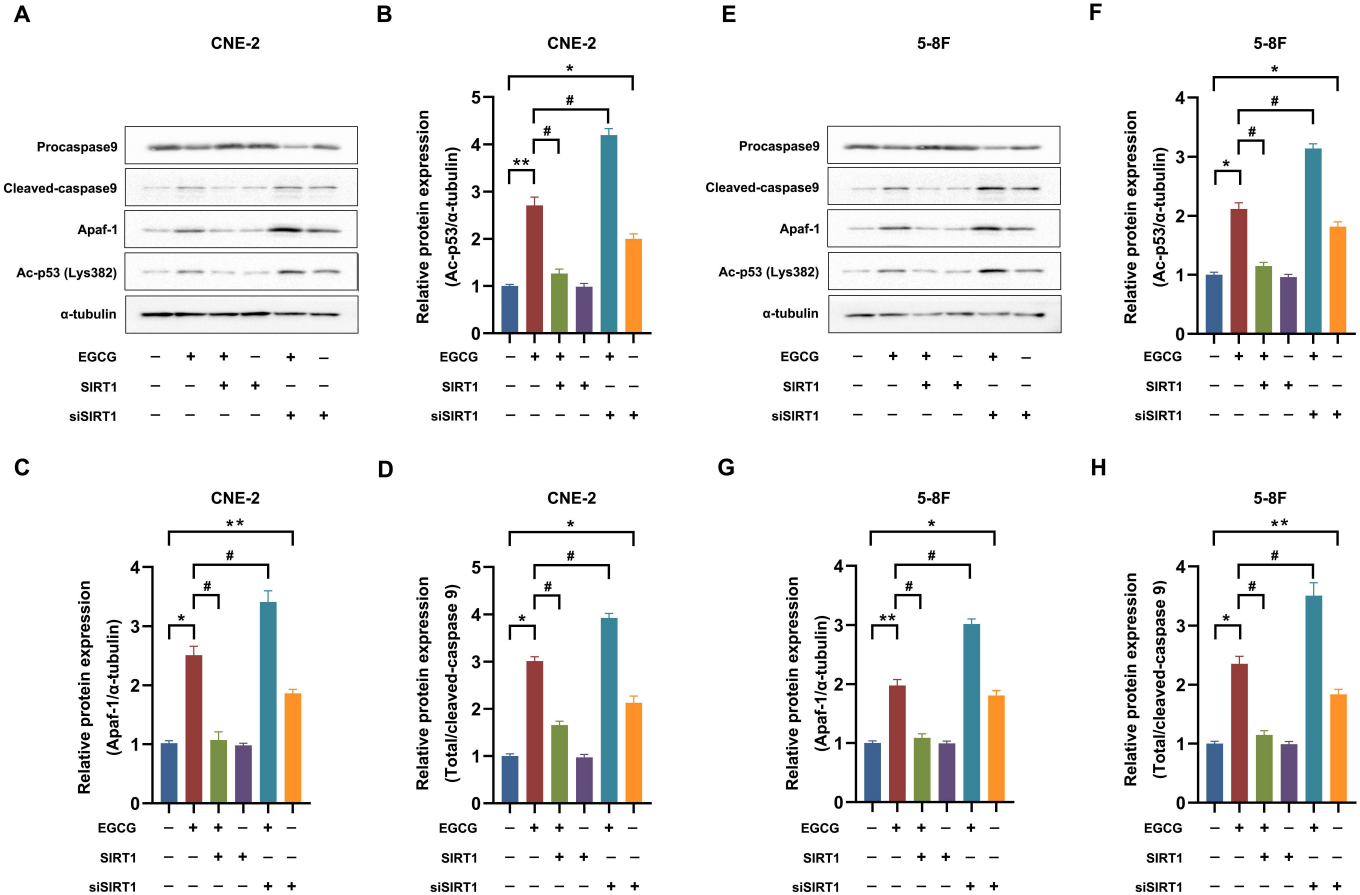
Effect of EGCG on p53-dependent apoptotic pathways in NPC Cell Lines. Cells transfected with or without SIRT1/siSIRT1 were treated with 40 μM EGCG for 48 h. The protein expression of Ac-p53, Apaf-1, and caspase 9 was detected by Western Blotting in CNE-2 **(A–D)** and 5-8F **(E–H)** cell lines. *P < 0.05 and **P < 0.01 vs. the group without treatment, ^#^*P* < 0.05 *vs*. the group without treatment with EGCG alone, *n* = 5.

**Figure 7:**
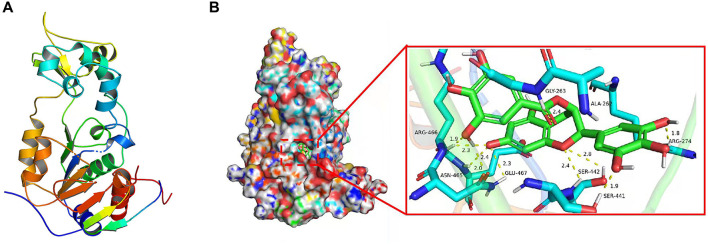
EGCG was docked to the SIRT1 protein structure. **(A)** The protein structure of SIRT1. **(B)** Molecular docking between EGCG and SIRT1.

**Figure 8:**
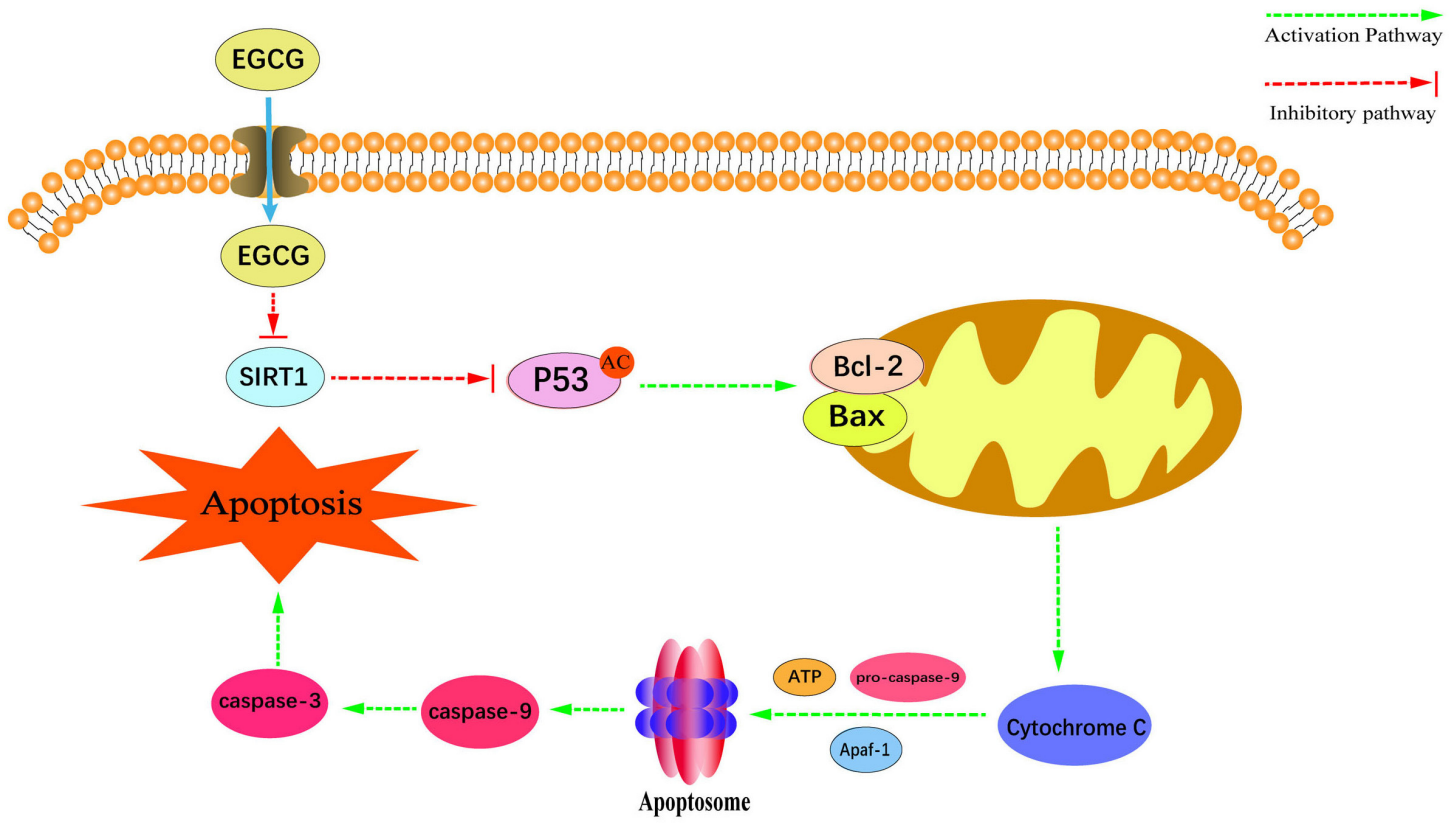
Schematic diagram of the proposed mechanisms of EGCG-induced apoptosis and anti-proliferation in NPC cell lines.

Previous studies showed that SIRT1 might deacetylate p53, thereby inverting p53-mediated cell growth arrest and apoptosis in many cancer cells (18). So, the effect of EGCG on p53-dependent apoptotic pathways in the NPC cells was studied. As shown in Figure 6, EGCG treatment could significantly increase the protein expression of Ac-p53, Apaf-1, and cleaved-caspase 9, which could be attenuated by preincubation with SIRT1 overexpression. However, the knockdown of SIRT1 aggravated the level of these proteins in CNE-2 (Figures 6A–D) and 5-8F (Figures 6E–H) cells. These results suggested that SIRT1 may participate in the effect of EGCG on the protein expression of p53-dependent apoptotic pathways.

### Molecular Docking Study Analyzed the Interaction Between EGCG With SIRT1

To investigate the mechanism by which EGCG-inhibited SIRT1, molecular docking was performed using Discovery Studio 3.1 software. We first obtained the SIRT1 crystal structures from the Protein Data Bank (PDB: 4KXQ) (Figure 7A). As shown in Figure 7B, the binging mode of EGCG and crystal structure of SIRT1 showed that EGCG enters the pocket of SIRT1 by seven hydrogen bonds, which turns out to be ARG-466 (1.9 Å), ASN-465 (2.4 Å), GLU-467 (2.3 Å), SER-442 (2.4 Å), SER-441 (1.9 Å), ARG-274 (1.8 Å), and GLY-263 (2.4 Å). ARG-274 and ARG-466 have been reported as the critical amino acids affecting SIRT1 activity, which suggested the regulatory effect of EGCG on the deacetylase activity of SIRT1 may be related to its interaction with SIRT1.

## Discussion

Nasopharyngeal carcinoma is the most prevalent malignant tumor in China, characterized by insidious location, lack of obvious early symptoms, and poor specificity (19–21). Currently, radiotherapy combined with chemotherapies is considered the standard treatment for NPCs (22). However, NPC patients often have a poor prognosis following treatment due to specific toxicities and drug resistance against chemotherapeutic drugs (23). Hence, it is urgent to exploit highly efficient and low-toxicity anticancer drugs combined with radiotherapy to improve the therapeutic effect of NPC.

Epidemiological studies have shown that the consumption of green tea is inversely associated with the incidence of certain cancers, and long-term consumption of green tea may reduce the risk of developing cancer and metastasis (24, 25). Green tea contains polyphenolic compounds, including flavanols, flavanonols, flavonoids, and phenolic acids accounting for almost 30% of the dry weight of green tea leaves. Most of the polyphenols in green tea are flavanols commonly known as catechins (26). EGCG, the major catechins in green tea, shows multiple biological activities such as antibacterial, antiviral, cardiovascular protection, and antiangiogenesis (7). Currently, the anti-cancer potential of EGCG has gained much attention on a global scale. Studies have shown that EGCG may intervene in the occurrence and development of cancers by inhibiting cell proliferation, inducing apoptosis, interference in cellular metabolism, inhibiting oncogene expression, and inhibiting tumor neovascularization (27). In a clinical study on subjects given EGCG at a single dose of 1,600 mg or 800 mg a day for 1 month, no obvious toxicity or side effects were seen except mild gastrointestinal reactions (28, 29). However, some animal studies reported the adverse effects linked with the consumption of high doses of EGCG. Moreover, an oral administration of 2,000 mg/kg, e.g., Teavigo, to rats resulted in about 80% mortalities, and histological analysis revealed hemorrhagic lesions in the stomach and intestine (30). In addition, high oral doses of EGCG (2,000 mg/kg) have also been reported to induce hepatotoxicity *in vivo* mice models (31). Despite this, the high doses of EGCG used in these articles have far exceeded the conventional doses used in animals and humans, suggesting the safety of EGCG. The EGCG significantly inhibited proliferation by inducing apoptosis in NPC cells. These results indicated that EGCG might be a potential chemotherapeutic agent for the treatment of NPCs. However, the exact molecular mechanism of EGCG-mediated inhibition of proliferation in NPC cells is not well elucidated.

A recent study has shown that EGCG could inhibit the proliferation of the nasopharyngeal CNE-1 cell line by inhibiting NF-κB nuclear translocation and EGFR phosphorylation (32). Moreover, EGCG has also been reported to inhibit the migration of the HONE-1 cell line by inhibiting the expression of MMP-2 (33). In addition, EGCG also inhibits NPC cells' invasion by regulating miRNA-296 (34). However, the mechanism of EGCG regulating the proliferation of NPC cells is still poorly known; hence, it is necessary to explore the precise molecular mechanism of EGCG inhibition of nasopharyngeal carcinoma cell growth. Sirtuins are a family of NAD^+^-dependent deacetylases involved in the multiple biological processes, including cell survival, DNA damage/repair, life span, and aging (35). There are seven different sirtuins in mammals, namely, SIRT1-SIRT7 (36). Recently, the sirtuin family has attracted much attention in cancer research, as they play an essential role in the onset and progression of cancer (37). SIRT1, the most extensively characterized family member, has also been demonstrated to be involved in cancer progression (38, 39). However, SIRT1 can function as both a tumor promoter and tumor suppressor simultaneously, depending on the immediate microenvironment (40). On the one hand, SIRT1 inhibits tumor formation by inhibiting tumor promoters such as NF-κB and c-Myc (41, 42). Furthermore, SIRT1 may suppress tumor cell apoptosis by inhibiting tumor suppressor genes such as P53, FOXO1, and FOXO3 (43, 44). Among them, the first discovered non-histone target of SIRT1, the p53 is suggested to play a central role in SIRT1-mediated functions in tumorigenesis (18). SIRT1 physically interacts with p53 and deacetylates p53 K382, inhibiting p53 activity, thus enabling cells to bypass p53-mediated apoptosis help the cells survive (43). In both of the NPC (CNE-2 and 5-8F) cell lines, the expression of SIRT1 were significantly increased compared with normal nasopharyngeal epithelial cells NP69, and SIRT1 overexpression could promote the proliferation and migration of NPC cells (14). In addition, SIRT1 is a direct target of miR-34a, and overexpression of miR-34a could increase the radiotherapy sensitivity of nasopharyngeal CNE-1 cells by inhibiting SIRT1 (45). These results suggest that SIRT1 plays a vital role in the developing and progression of NPCs. However, whether EGCG inhibits NPC cells' proliferation by regulating SIRT1 has not been reported. Herein, it is demonstrated for the first time that EGCG could inhibit protein expression and enzyme activity of SIRT1 in CNE-2 and 5-8F cells in a dose-dependent manner. Further studies showed that SIRT1-p53 signaling participated in the effect of EGCG on NPC cells.

Although it was also found that EGCG induces apoptosis in NPC cells through SIRT1 inhibition, however, whether SIRT1 acts as an oncogene or tumor suppressor may depend on the stages of tumor development or upstream and downstream regulators. In some cases, whether activation of SIRT1 also could inhibit the growth of NPC cells is still worth exploring. Moreover, initially, the inhibitory effect of EGCG on SIRT1 was not specific and displayed different modalities of regulation in different cell lines. The research showed that EGCG inhibits homocysteine-induced oxidative damage in endothelial cells by activating the SIRT1/AMPK pathway (46). The study also demonstrated that EGCG inhibits hepatic cholesterol synthesis by targeting SREBP-2 through modulation of the SIRT1/FOXO1 signaling pathway (47). Our previous work showed that EGCG inhibited the growth of H9C2 cardiomyocytes by suppressing the expression of SIRT1 (15). In this study, we are the pioneers to report that EGCG-induced apoptosis in nasopharyngeal carcinoma CNE-2 cells by inhibiting the expression and activity of SIRT1. In addition to SIRT1, EGCG also affected other Sirtuin family proteins. For example, previous EGCG could regulate senescence and anti-SASP *via* SIRT3 in 3T3-L1 Preadipocytes (48). Moreover, previously we reported that EGCG increased SIRT6 activity by affecting the level of NAD (16). The regulatory role of EGCG on other members of the Sirtuins family needs to be further investigated.

How does EGCG carry out its inhibitory regulatory role on the expression of SIRT1 or not? Following the literature, it is shown that the regulation mode of SIRT1 by EGCG remained variable in the different cell lines. It was demonstrated that EGCG could activate SIRT1 in endothelial cells (46), while our previous study also showed that EGCG could inhibit SIRT1 expression in H9C2 cardiomyocytes (15). This work demonstrated that EGCG inhibited proliferation and induced apoptosis in NPC CNE-2 cells by downregulating SIRT1. However, the specific molecular mechanism by which EGCG regulates SIRT1 expression in NPC cells is still unknown. To clarify the regulatory mechanism of EGCG on SIRT1, at first, the effect of EGCG on the mRNA expression of SIRT1 was examined, and no impact on the mRNA level of SIRT1 was observed, suggesting that EGCG does not inhibit the expression and activity of SIRT1 at the transcriptional level (Supplementary Figure S3). Therefore, the interaction between EGCG and SIRT1 using a molecular docking approach to clarify EGCG was the only molecule to bind directly to SIRT1 (Figure 7). However, the interaction between EGCG and SIRT1 needs to be further justified through surface plasmon resonance (SPR) methods. Moreover, whether the binding of EGCG to SIRT1 affects the expression and activity of SIRT1 needs to be further investigated.

In conclusion, this study has demonstrated for the first time that EGCG could activate the mitochondrial apoptotic pathway by inhibiting the SIRT1-p53 signaling pathway and finally inducing the apoptosis in human NPC cell lines (Figure 8). Our study further elucidated the critical role of SIRT1 in the occurrence and development of NPC, which provided the necessary experimental data and theoretical basis for the application of EGCG in the prevention and treatment of NPCs.

## Data Availability Statement

The original contributions presented in the study are included in the article/supplementary material, further inquiries can be directed to the corresponding authors.

## Author Contributions

SJ: investigation, data analysis, and writing—original draft. CH: investigation and writing—original draft. GZ, WY, BW, JT, and ML: resources and investigation. XL and BH: investigation. DW: data analysis. TY: funding acquisition, resources, and supervision. CW: writing—review and editing. YC: funding acquisition, conceptualization, supervision, resources, and writing—review and editing. All authors have read and agreed to the final manuscript draft.

## Funding

This work was supported by grants from the National Natural Science Foundation of China (Nos. 31972889 and 81300085), the Natural Science of Guangdong Province (No. 2017A030313571), and the High-level University Construction Fund of Guangdong Province (Nos. 06-410-2107240 and 06-410-2107244).

## Conflict of Interest

The authors declare that the research was conducted in the absence of any commercial or financial relationships that could be construed as a potential conflict of interest.

## Publisher's Note

All claims expressed in this article are solely those of the authors and do not necessarily represent those of their affiliated organizations, or those of the publisher, the editors and the reviewers. Any product that may be evaluated in this article, or claim that may be made by its manufacturer, is not guaranteed or endorsed by the publisher.
