# Supplementary material for: EGCG Inhibits Proliferation and Induces Apoptosis Through Downregulation of SIRT1 in Nasopharyngeal Carcinoma Cells
Source: Front Nutr. 2022 Apr 25;9:851972. doi: 10.3389/fnut.2022.851972 (PMC9084317; doi:10.3389/fnut.2022.851972)
Supplement: Supplementary file 1 [file Data_Sheet_1.docx]

**EGCG Inhibits Proliferation and Induces Apoptosis through downregulation of SIRT1 in Nasopharyngeal Carcinoma Cells**

**Supplementary Table S1**

Table S1. Primer sequences for qRT-PCR

| Primer | Sequences |
| --- | --- |
| SIRT1 | Forward:5'- TACCCAGAACATAGACACG -3' |
|  | Reverse: 5'- GTATTTACAAATCAGGCAAG-3' |
| BAX | Forward:5'- GAGACTCCTCAAGCCTCCTC -3' |
|  | Reverse: 5'- CACTGTGACCTGCTCCAGAA -3' |
| BCL-2 | Forward: 5'- TGTGGCCTTCTTTGAGTTCG -3' |
| GAPDH | Reverse: 5'- CTACCCAGCCTCCGTTATCC -3'  Forward: 5'- AGGAGTAAGAAACCCTGGAC -3'  Reverse: 5'- CTGGGATGGAATTGTGAG -3' |

**Supplementary Table S2**

Table 2. The siRNA sequences for SIRT1

| siRNA | Sequences |
| --- | --- |
| siSIRT1-1 | Sense:5'- UUCUGAAAUAUUCAAUAUCAA -3' |
|  | Antisense:5'- GAUAUUGAAUAUUUCAGAAAA -3' |
| siSIRT1-2 | Sense:5'- UUUGCAAACUUGAAGAAUGGU -3' |
| siSIRT1-3 | Antisense:5'- CAUUCUUCAAGUUUGCAAAGG -3'  Sense:5'- UAUGUUCUGGGUAUAGUUGCG -3'  Antisense:5'- CAACUAUACCCAGAACAUAGA -3' |
| Negative | Sense:5'- GCAUCUCCCUAGACCGAUUTT -3' |
|  | Antisense:5'- AAUCGGUCUAGGGAGAUGCTT -3' |

**Supplementary Figure S1**

**
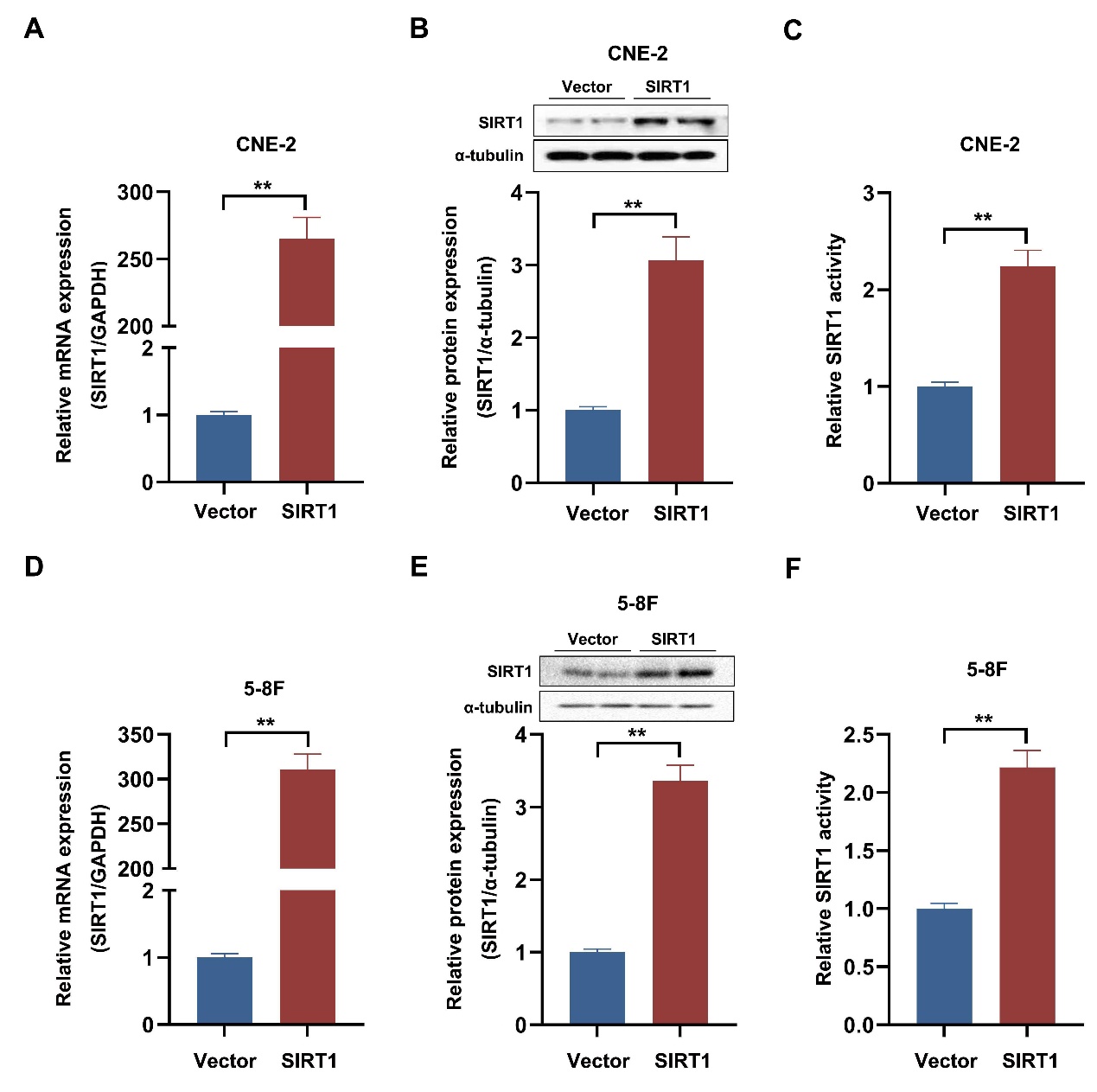
**

**Fig. S1 Effect of SIRT1 overexpression on the expression and activity of SIRT1.** CNE-2 and 5-8F cells were transfected with vector and Flag-SIRT1 respectively. The mRNA (A and D), protein (B and E) and enzyme activity (C and F) of SIRT1 were detected. ***P* < 0.01 *vs.* the Vector group, *n*=5.

**Supplementary Figure S2**


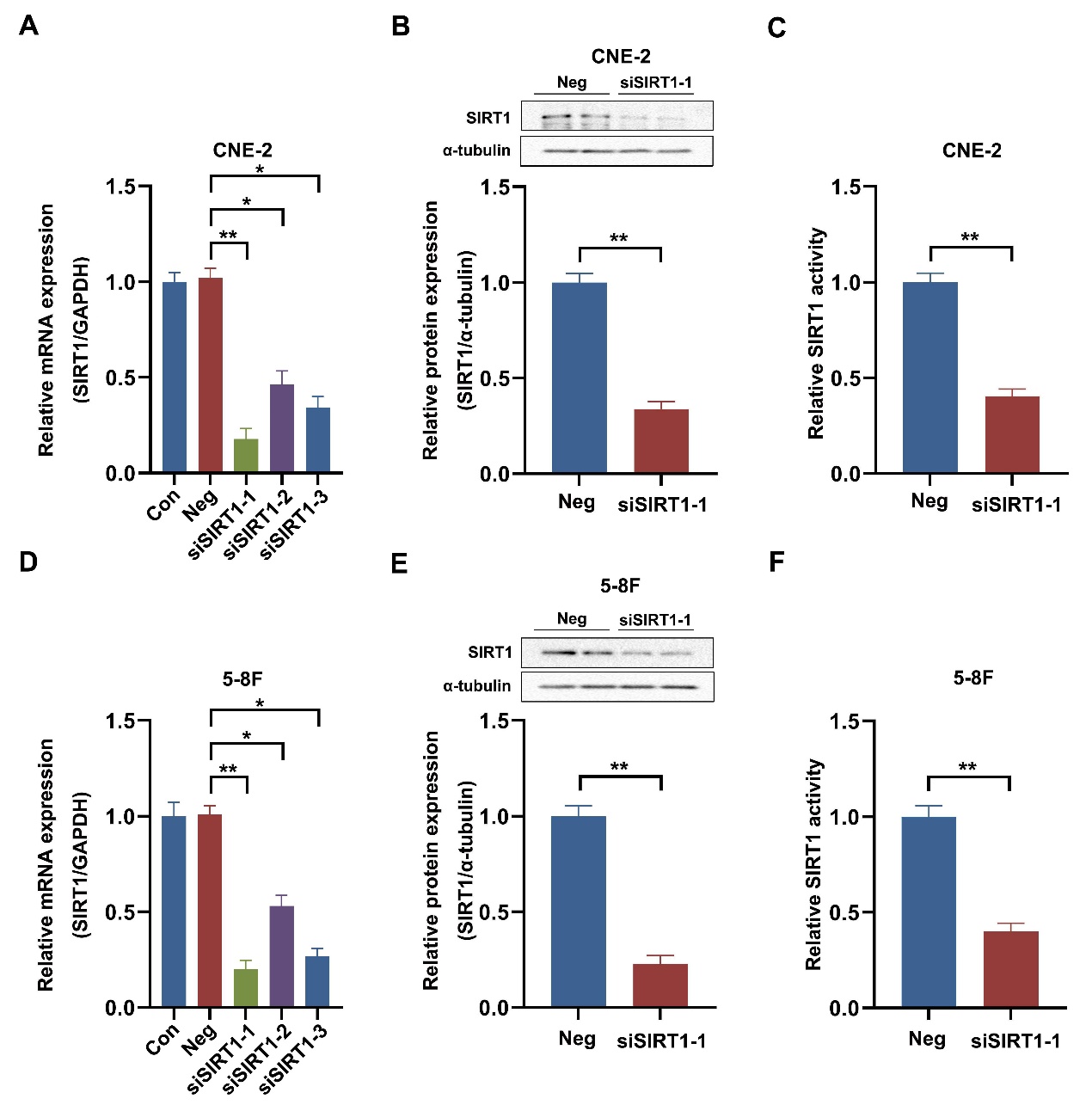


**Fig. S2 Effect of SIRT1 siRNA interference on the expression and activity of SIRT1.** CNE-2 and 5-8F cells were transfected with siSIRT1 (siSIRT1-1, siSIRT1-2, siSIRT1-3) and negative control siRNA respectively. The mRNA (A and D), protein (B and E) and enzyme activity (C and F) of SIRT1 were detected. **P* < 0.05 and ***P* < 0.01 *vs.* the Neg group, *n*=5.

**Supplementary Figure S3**

**
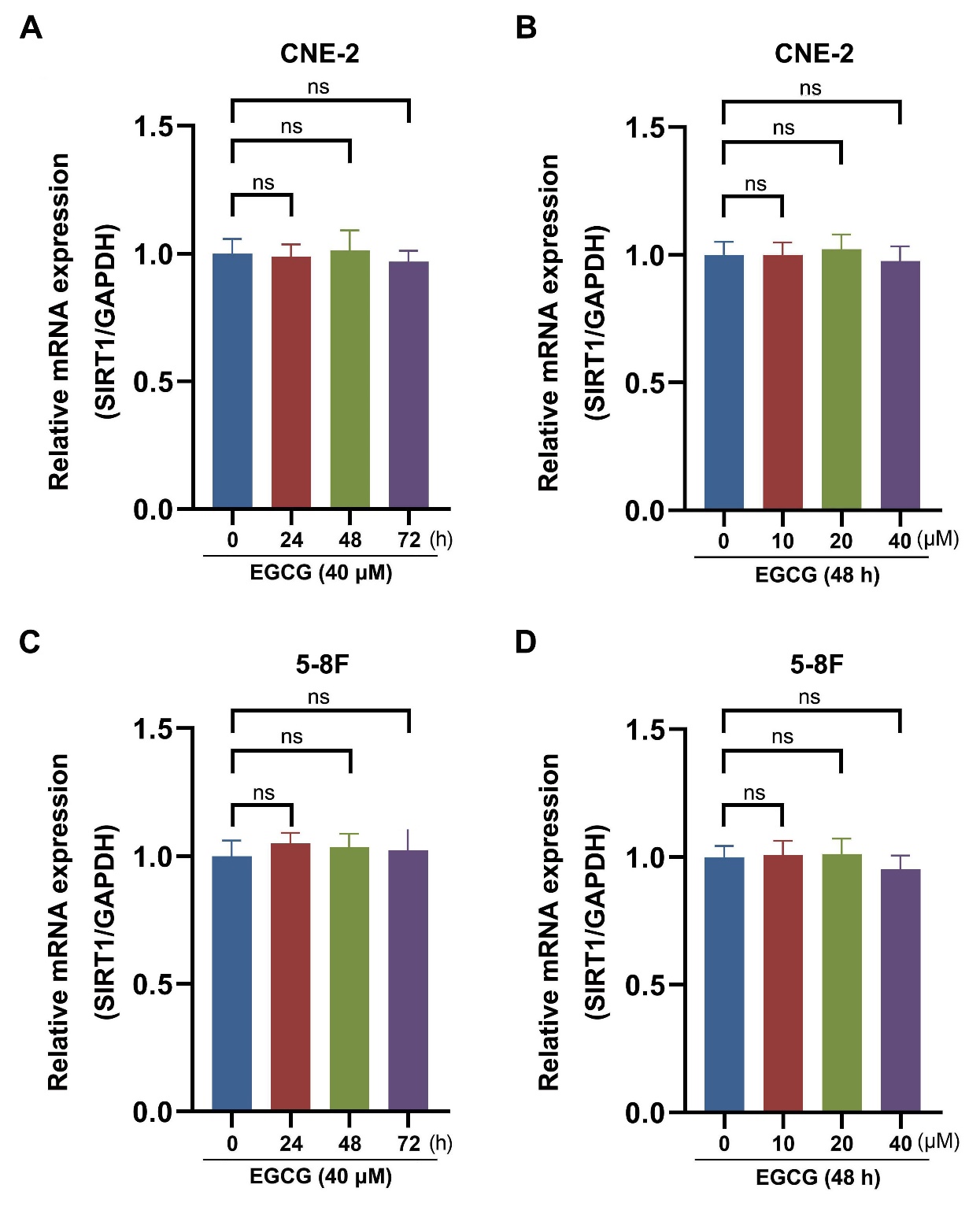
**

**Fig. S3 Effect of EGCG on mRNA expression of SIRT1 in NPC cell lines.** CNE-2 (A and B) and 5-8F (C and D) cells were treated with various concentrations of EGCG for 48 h or with 40 μM EGCG for indicated time points. The mRNA expression of SIRT1 were detected by Real-time PCR.
